# HCS—hierarchical algorithm for simulation of omics datasets

**DOI:** 10.1093/bioinformatics/btae392

**Published:** 2024-09-04

**Authors:** Piotr Stomma, Witold R Rudnicki

**Affiliations:** Faculty of Computer Science, University of Białystok, Białystok 15-245, Poland; Computational Centre, University of Białystok, Białystok 15-245, Poland; Faculty of Computer Science, University of Białystok, Białystok 15-245, Poland; Computational Centre, University of Białystok, Białystok 15-245, Poland

## Abstract

**Motivation:**

Analysis of the omics data with the help of machine learning (ML) methods is limited by small sample sizes and a large number of variables. One possible approach to deal with such data is using algorithms for feature selection and reducing the dataset to include only those variables that are related to the studied phenomena. Existing simulators of the omics data were mostly developed with the goal of improving the methods for generations of high-quality data, that correspond with the highest possible fidelity to the real level of molecular markers in the biological materials. The current study aims to simulate the data on a higher level of generalization. Such datasets can then be used to perform tests of the feature selection and ML algorithms on systems that have structures mimicking those of real data, but where the ground truth may be implanted by design. They can also be used to generate contrast variables with the desired correlation structure for the feature selection.

**Results:**

We proposed the algorithm for the reconstruction of the omic dataset that, with high fidelity, preserves the correlation structure of the original data with a reduced number of parameters. It is based on the hierarchical clustering of variables and uses principal components of the clusters. It reproduces well topological descriptors of the correlation structure. The correlation structure of the principal components of the clusters then is used to obtain datasets with correlation structures similar to the original data but not correlated with the original variables.

**Availability and implementation:**

The code and data is available at: https://github.com/p100mma/hcrs_omics.

## 1 Introduction

Modern methods of molecular biology, collectively known as *omics* generate data on biological processes with very high resolution—such as, e.g. expression levels of individual genes in the individual cells. The datasets obtained with such methods help researchers elucidate the mechanisms of cancer or genetic diseases. Due to the very high cost of experimental studies, and legal and ethical concerns, the sample sizes arising in such experiments are most often small, whereas the number of variables can reach hundreds of thousands. Therefore, the analysis of such data often involves the use of specially designed protocols that utilize feature selection and machine learning (ML) algorithms. Validation of such protocols is crucial, especially when one deals with black-box ML algorithms. One of the possible approaches to validate them is to test them on datasets with properties similar to the experimental ones, but where the ground truth is known, hence the true performance can be measured. To this end, generated data should have statistical properties (known *a priori*) resembling the ones of reference datasets. Multiple tools for the simulation of read distribution in the single sample have been developed, in particular for the single cell transcriptomics, the detailed review of the field is given in the article by Sun and coworkers in an article introducing scDesign2 tool (Sun *et al.* 2021). They aim at the generation of datasets that can be used as a benchmark for analysis of the single cell data. To our knowledge, the only tool available for simulations of transcription for the bulk data is the WGCNA package (Zhang and Horvath 2005). However, its primary goal is the analysis of gene expression, and simulations are of secondary importance. The structure of simulated datasets doesn’t reflect well the complex correlation structure of gene expression, see Figs 2–4.

The classical method for generating data correlated according to a known covariance matrix Σ uses Cholesky Decomposition (Press *et al.* 2007), which requires Σ to be positive definite (PD) (Higham 2009). When the number of samples is larger than the number of variables, the estimate of Σ is not PD. There are methods of dealing with this problem, such as “repairing” techniques that can find the nearest PD approximation (Higham 2002), but these methods have problems with the interpretability of the results. However, problems of interpretability as well as estimation in the presence of high-dimensionality and noise can be dealt with using unsupervised approaches.

Simulating marginal distributions of genes alone is much easier. Techniques such as inverse transform sampling (Press *et al.* 2007) can be used along with parametric distribution fitting, to generate new random variates which replicate reference one-dimensional distributions. Coupling probability integral transform with quantile function transformations (inverse transform) can be used to estimate both marginal distributions and latent covariance matrix, as was done in Sun *et al.* (2021).

### 1.1 Clustering-based approaches in biology

Clustering can be used either for purposes of grouping samples into sub-types (like deriving cell-types in single-cell sequencing Xu and Su 2015, Stuart *et al.* 2019) or for grouping genes themselves, into highly correlated clusters, for purposes of identifying gene “modules” (D’haeseleer 2005, Zhang and Horvath 2005). For example, in the context of genome-wide gene expression profiling, modules are groups of genes with similar expression profiles, often co-regulated and functionally related (Saelens *et al.* 2018). While some concerns about finding modules by clustering methods have been raised (Saelens *et al.* 2018), correlation-based clustering methods are still widely used to infer new knowledge from experimental data in biomedical applications—co-expressed genes typically are involved in the same processes (van Dam *et al.* 2012) and therefore—clusters of genes derived from data alone can be used to find relevant biomarkers (Tian *et al.* 2020, Feng *et al.* 2022). Gene Ontology Enrichment (Huang *et al.* 2009) is often another step in the analysis, and clustering that produces better enrichment scores tends to be preferred by researchers (Song *et al.* 2012).

### 1.2 Weighted graph clustering

A graph model of data, in which entities are nodes in a graph (network), is often preferred for the analysis of biological data. Grouping is then done based on the topological characteristics of the graph. A model of a weighted network also encodes the strength (and possibly the sign) of the relations between nodes. Numerous approaches to the problem exist, for details, we refer to Fortunato (2010) and Fortunato and Hric (2016). Notable examples include (Rosvall and Bergstrom 2008, Van Dongen 2008), some have found use in the biological domain (Shih and Parthasarathy 2012, Stuart *et al.* 2019). (Dis)similarity-based methods, such as classical bottom-up hierarchical clustering algorithm, can also be applied under the graph model (Fortunato 2010).

Two methods which we use in this research are the Markov Clustering Algorithm (MCL) (Van Dongen 2008) and WGCNA framework (Zhang and Horvath 2005, Langfelder and Horvath 2009). MCL finds clusters from an equilibrium state of an enhanced random walk, in which stronger probabilities are amplified (by a process called inflation). In WGCNA, one uses a transformed value of correlation coefficient to group variables (typically genes) into clusters interpreted as “modules.” Apart from a robust clustering protocol, WGCNA offers methods of analysis especially suited for networks derived from correlation (Zhang and Horvath 2005). WGCNA also uses cluster-wise Principal Component Analysis (PCA) to derive an “Eigengene,” a latent factor summarizing the characteristics of the correlated genes in the module (Langfelder and Horvath 2009).

### 1.3 PCA in the low sample, high variable setting

In classical PCA (Jolliffe 2002), one finds coordinates of the data in a different set of axes, where the first axes carry most of the overall variance. Each *PC* depends on each original variable in the dataset, which is not ideal for interpretation in gene expression data. This has led to the development of sparse PCA methods (Zou *et al.* 2006). WGCNA deals with the problem by performing PCA cluster-wise.

By using only *k* PCs in the decomposition, we can obtain an approximation to the original dataset, which is not independent of it (Halko *et al.* 2011). However, including some randomization in the process can lead to a basic simulation method of the data. A simple heuristic similar to the method just described exists in WGCNA R (Langfelder and Horvath 2009) package, which can be used for simulating data with correlation described by input clustering. The aim of this article is to develop an improved simulation procedure that also possesses the intuitiveness of the original WGCNA approach, but can produce much more realistic results.

## 2 Materials and methods

As in WGCNA methodology, we propose to utilize a correlation based clustering of variables and to perform *PCA* in each cluster separately. This is consistent with the intuition that a set of highly correlated variables can be accurately described by a structure of lower dimensionality. Such decomposition requires using more *PC*s than a classic approach, but it better reflects complicated correlation structure in the data.

To explain a fraction total variance in the dataset, we could explain a fraction of the total variance of each cluster. If the clustering we use is really fine-grained, then the required number of *PC*s to use in each cluster to reach the target variance can be satisfactorily small. However, a large number of clusters is still difficult to interpret, if we do not know at least if they are related in some manner. That’s why we propose a hierarchical approach.

**Figure 1.**
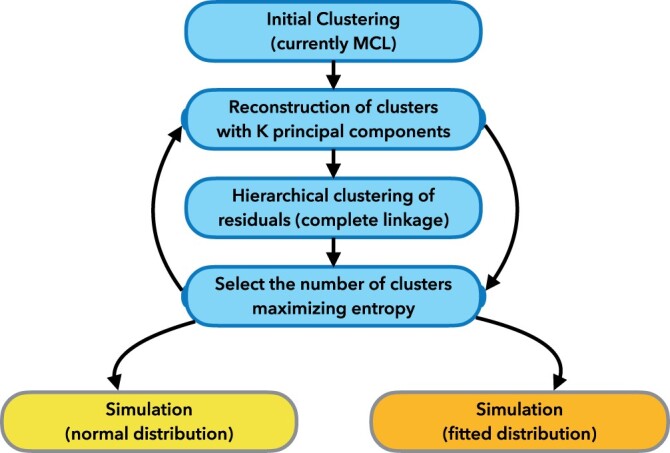
Schematic representation of HCR algorithm (four boxes on top) and HCS simulations (two bottom boxes).

The general procedure for the simulation is described in Fig. 1.

### 2.1 Definitions and notation

We assume that dataset is described by set of *N* variables X, each *X_i_* determined by the set of indexes I: $X=\left\{X_{i}|i\in I\right\}$. We define clustering $P=\{C_{1},C_{2}\ldots \}$ as a partition of indexes: $I=\cup_{i}C_{i}$, where for $i\neq j:C_{i}\cap C_{j}=\emptyset$. Hierarchical clustering is a sequence of (nested) clusterings, in which the sets of the $P_{l+1}$ subdivide clusters of the previous clustering *P_l_*. $V_{C_{i}}^{X}$ will mean sum of variances of variables *X_j_* from X with $j\in C_{i}$, i.e. $V_{C_{i}}^{X}=\sum_{j\in C_{i}}V\text{ar}(X_{j})$. If we omit the subset of indexes in the notation, like $V^{X}$, that means the sum runs over all elements of X.

Consider now set of *PC*s $PC_{1},PC_{2}\ldots PC_{M}$ of X, which were calculated separately for each cluster in *P*. Note that in contrast to original PCA, $\text{Var}\;(\sum PC_{i})\;\neq \;\sum \text{Var}\;(PC_{i})$, since *PC*s of different clusters might be correlated. However, such property holds locally in each cluster:

(1) $$V^{X}=\sum_{C_{j}\in P}V_{C_{j}}^{X}=\sum_{C_{j}\in P}\;\text{Var}(\sum_{l}PC_{jl}^{X}).$$

Nevertheless, one can reconstruct each variable *X_i_* by using only *k PC*s of the cluster to which it belongs. We will call such reconstruction a *k*th order reconstruction of X based on *P*.

In the HCR algorithm, we extend this construction to be compatible with hierarchical clustering. The algorithm uses several mathematical facts, which make it work. See the Supplementary Material for derivations.

### 2.2 Hierarchical clustering based reconstruction algorithm—HCR

We describe the HCR algorithm, for reconstructing data based on PCA combined with hierarchical clustering. The main input parameters are: limiting fraction of variance to explain: $f_{E}\in [0,1]$, *k—*maximum number of *PC*s to use per cluster.

The algorithm uses two methods for clustering variables based on their correlation: an initial “adaptive” clustering algorithm and a “separator” algorithm. These can be any methods that satisfy the following criteria. The initial “adaptive” algorithm should be able to detect clusters of various sizes, and preferably be able to perform initial prefiltering: it should classify some variables to a “noise” subset, denoted as *C*_0_ (not real clusters), irrelevant to the overall correlation structure of the rest of the network (noiseless part, union of “proper” clusters, denoted $C_{i\neq 0}$). *C*_0_ could be variables distant from every other variable or low variance ones. In contrast, a “separator” should produce a complete partition of the set of variables.

“Adaptive” algorithm produces the first partition *P*_1_. The “separator” algorithm is used to produce further subdivisions $P_{2},P_{3}\ldots$ of initial clusters, based on correlation of the “residuals.”

The general steps of the HCR algorithm are then:

1. Initial division of X by an adaptive clustering algorithm, producing partition *P*_1_. If $f_{E}V^{X}>V_{C_{i\neq 0}}^{X}$, stop—there is not enough variance in the noiseless part of the dataset for given *f_E_*. Otherwise, calculate $X^{1}$—*k*th order reconstruction of X based on *P*_1_.
2. For each $C_{j}\in P_{1}$ with unexplained variance left, subdivide it based on residuals $E^{1}=\{X_{i}-X_{i}^{1}\}$ in *C_j_*; after processing each *C_j_*, produce *P*_2_ and $X^{2}$, a *k*th order reconstruction of residuals $E^{1}$ based on *P*_2_. Calculate $E^{2}=\{X_{i}-X_{i}^{1}-X_{i}^{2}\}$.
3. Continue subdivisions of clusters with unexplained variance until convergence of the reconstruction is satisfactory.

The exact technical description of a generalized version of the algorithm is moved to Supplementary Material. Here we provide a general overview and some practical remarks. The final reconstruction of each *X_i_* is set to $X_{i}^{R}:=\sum_{j=1}^{g}X_{i}^{j}$, where *g* is the number of levels of hierarchy used. Each $X_{i}^{R}$ is a linear combination of at most *g *×* k PC*s of clusters to which *i* belongs. If *k *=* *5, *g *=* *2, *P*_1_ contains 3 clusters, and *P*_2_ 11, assuming no cluster was explained in *P*_1_ fully, this gives 70 *PC*s in total, 5 per (sub)cluster. However, each reconstructed variable is a linear combination of at most 10 (5 + 5) generating *PC*s (which are independent). The set of all *PC*s and coefficients used to reproduce variables in HCR is further referred to as a hierarchical clustering-based decomposition (HCD) of the dataset X. In principle, it is always true for the reconstructed variable $X_{i}^{R}$ that (see Supplementary Material for derivation)

(2) $$\text{Var}(X_{i})=\text{Var}(X_{i}^{R})+\text{Var}(E_{i}^{g}),$$

so as an internal convergence criterion, we measure total variance explained as $V^{X^{R}}/V^{X}$.

The last step (continue until exhaustion) can be too excessive in practice. Our experiments on real data suggest that even after two to three levels of clustering, the remaining signal can be regarded as noise. Therefore, we recommend applying a fixed number of subdivision steps, then examining the results and eventually continuing further based on that.

Currently, for a “separator” algorithm, we use hierarchical clustering based on the complete linkage method (with initial distance between residuals *i*, *j* calculated as $1-\text{cor}{\left(E_{i},E_{j}\right)}^{2}$). The best split is chosen from several candidate ones by maximizing the normalized entropy of a division: $\left(-\sum_{i=1}^{n_{g}}p_{i}\;\text{log}\;p_{i})/(\text{log}\;n_{g}\right)$. *n_g_* is the number of clusters in the split, *i* ranges over different subclusters in the split, and *p_i_* is the proportion of the subcluster in the whole split. This quantity is the biggest for an even split. This combination deals with the empirically observed tendency of the clustering algorithms, which tend to split initial clusters into one big sub-cluster and periphery, gathered into much smaller sub-clusters.

HCR is used to model a latent correlation structure of really high-dimensional datasets but is not a simulation. However, it can be used to obtain a simulation method with several desirable properties:

1. it realistically replicates correlations of the original dataset to the known degree of accuracy,
2. it learns the general structure of real data, and summarizes it with a smaller set of latent factors,
3. it produces variables with known parametric distributions,
4. it produces new, independent synthetic data samples with real-data-like *N*-variate distribution.

### 2.3 HCS simulation procedures

Each variable in *HCR* is a lin. combination of *PC*s: $X_{j}^{R}=\sum_{i}\beta_{ji}PC_{i}$. In *HCS* method, to simulate variable $X_{j}^{S}$, we use same equation but replace original *PC*s with synthetic ones. Correlations between synthetic *PC*s are identical to correlations between original ones, but a synthetic set of *PC*s is independent of the original one.

We propose two simulation methods for producing variables with a similar correlation structure to the reference data. Both require HCD of real data as an input and model covariance of associated *PC*s. In the first one, each simulated variable follows a known normal distribution. We refer to it further as HCS(n). The second one, HCS(f), is an extension of the first one, where we also model marginal distributions of *PC*s. “f” stands for “fitted” distributions of *PC*s. HCS(f) requires a choice of a parametric family of distributions to model the associated *PC*s. Since shapes of distributions of *PC*s can be difficult to predict, we propose to use a metalog family of distributions (Keelin 2016), which are flexible, exhibit a closed form quantile function, are fit by a linear procedure and allow for modelling multimodal distributions (Keelin 2016).

**Figure 2.**
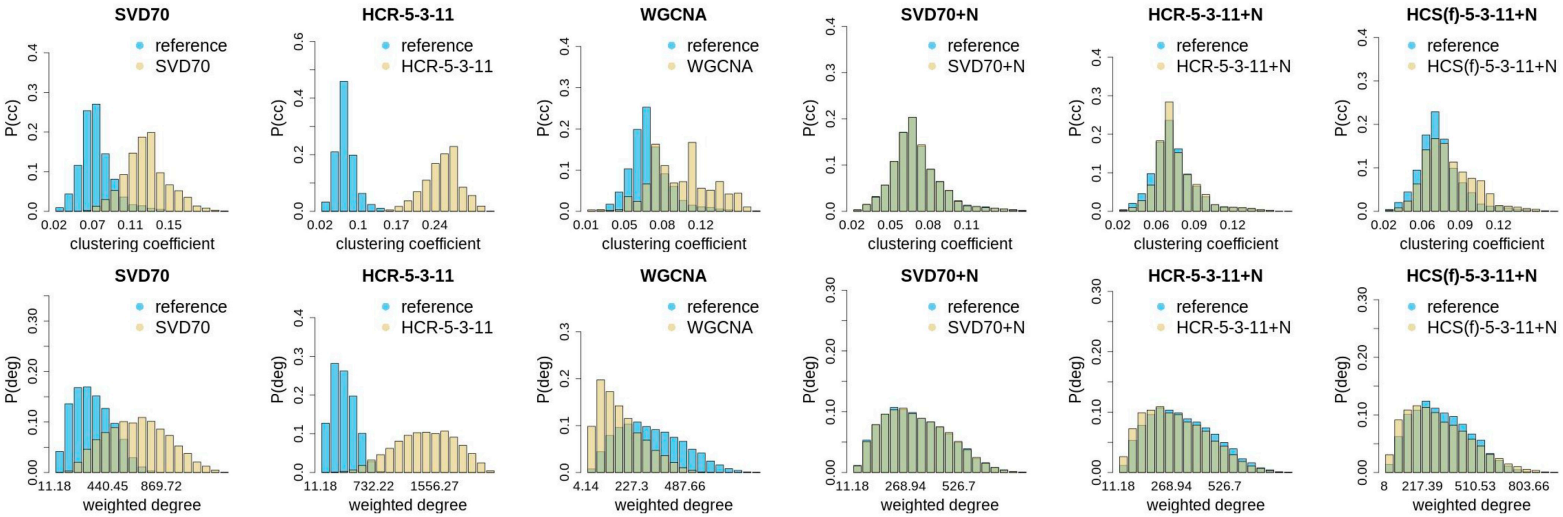
Clustering coefficient (top row) and weighted degree distributions (bottom row) for different methods of reconstruction and simulation of the BRCA dataset (yellow/brighter histograms). The distribution of the clustering coefficient in the original dataset (blue/darker histograms) is shown for comparison. The labels: SVD10 and SVD70 denote a reconstruction of the dataset with 10 and 70 singular vectors, respectively; HCR-X-Y-Z, HCS(n)-X-Y-Z, and HCS(f)-X-Y-Z correspond to the reconstruction with the HCR algorithm, simulation with the HCS algorithm using the normal distribution of principal components, and simulation with the HCS algorithm using the fitted distribution of principal components, respectively, parameters X, Y, and Z are the number of principal components used in reconstruction, the number of clusters at the first level of the hierarchy, and the number of clusters at the second level of the hierarchy, respectively. The +N label denotes that the noise was added to the reconstruction or simulation. Note that different scales for the *X*-axis are used in the individual plots.

**Figure 3.**
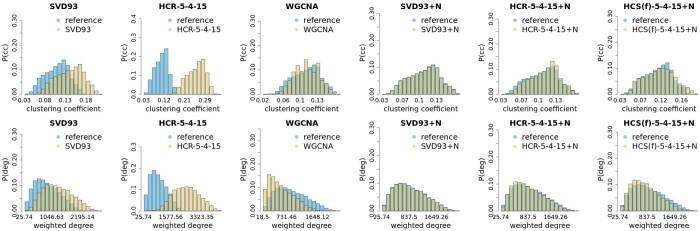
Weighted degree and clustering coefficient distributions for different methods of reconstruction and simulation of the KIRC dataset. The distribution for the original dataset is shown for comparison. The layout, labels, and colours are as in Fig. 2.

At the last step of HCS, an amount of random noise is added to each simulated variable, to make its variance equal to its real counterpart. This utilizes the relation in Equation (2) and is an important factor for making the simulation realistic (see Figs 2 and 3). If HCR is to be used as a faithful replication of real data, this step should also be included. We refer to the Supplementary Material for computational details of procedures.

### 2.4 Implementation

To investigate the performance of the proposed algorithm, we have run a series of computational experiments on high-dimensional gene expression data. As an initial test dataset, we used a dataset of RNA-seq gene expression profiles of breast cancer patients (BRCA) (Pereira *et al.* 2016). After initial prefiltering and preprocessing (as done in Polewko-Klim *et al.* 2021), the set consists of 1394 samples, described by 8673 gene expression profiles. This dataset contains arguably a larger number of samples than usually seen in typical applications. Therefore, we have run additional tests on a smaller (605 samples), well-known KIRC dataset from TCGA, containing highly correlated RNA-seq gene expression levels taken from kidney cancer study (Peng *et al.* 2015). After standard preprocessing (as in Polewko-Klim and Rudnicki 2020), for modelling, we used 15 166 genes with the highest variance.

All relevant code was written in R language and is available in the public repository (https://github.com/p100mma/hcrs_omics), along with mentioned preprocessed input.

In the first step, we compared our method with two reference approaches on BRCA data: SVD reconstruction and WGCNA simulation. For plain SVD-based reconstruction of real data without clustering, we have used 10 and 70 *PC*s. 70 corresponds to the total number of *PC*s we use in our method, and 10 is the number of *PC*s used in our method to replicate each variable. We also compare against SVD reconstruction with added noise as in the last step of the HCS procedure.

We have also adopted an initial simulation heuristic implemented in R WGCNA package (Langfelder and Horvath 2009), which also uses clustering as an input. For the basis of this simulation method, we have used a version of the standard clustering protocol proposed by WGCNA (Zhang and Horvath 2005), utilizing in the end stage publicly available R package: dynamicTreeCut (Langfelder *et al.* 2016). Since the original simulation function from the WGCNA R package was not suited for real simulation studies, we benchmark against a modified version.

For initial clustering in our method, we have chosen the MCL algorithm based on the square of Pearson correlation. We have used the original C implementation of the MCL algorithm made by its original author (Van Dongen 2008). Clusters significantly smaller than the rest were set as “noise.” In further divisions using the complete linkage method, we chose the best split out of splits into a number of clusters ranging from 2 to 7. Lastly, for fitting distributions in simulation method HCS(f), we utilized R package rmetalog (Faber and Jung 2021). For additional tests on the KIRC dataset, we have used a similar approach, utilizing two layers of hierarchy of clusters, maximal number of *PC*s per cluster set at 5, yielding 93 *PC*s used in total and 10 *PC*s used per variable. Details of the modified WGCNA simulation and the parameters of the methods used are described in the Supplementary Material.

## 3 Results

Several metrics were used for the analysis of results. The basic ones for the reconstructions are the fraction of variance of the original data explained by the reconstructions, the similarity between the original and reconstructed variables, and the difference between the original correlation structure of the data and the reconstruction. In the case of simulations, the only metric that should be carried over from the reconstructions is the difference between the simulated and real correlation structure. In contrast with reconstructions, the correlations between simulated and original variables should be indiscernible from the noise. However, the general topological properties of the correlation structure of both reconstructed and simulated variables should be very close to that of the original dataset.

The numerical metrics comparing the overall structure of HCR/HCS data to the reference, for varying numbers of *PC*s and levels of hierarchical reconstruction, are shown in Table 1. The first column differentiates between reconstruction and simulation types. Five next columns describe the parameters of the reconstruction/simulation, the seventh column shows the fraction of variance explained by the model, and in the last three columns, three following measures of the distance between the model and reference are shown: maximal and average of the squared correlation computed between all variables from the real data and simulations/reconstruction as well as RMSE computed between correlation matrices.

**Table 1.** Reconstruction accuracy for varying parameters of the model.

Type	*k*	*g*	*n*_C_	*n*_PC_	+*N*	*V*_E_	*R*_mx_	*R*_avg_	CR
**BRCA**									
R	2	1	3	6	0	0.27	0.86	0.09	0.44
R	2	2	14	28	0	0.37	0.94	0.08	0.33
R	2	1	3	6	1	0.27	0.74	0.02	0.12
R	2	2	14	28	1	0.37	0.87	0.03	0.09
R	3	1	3	9	1	0.32	0.78	0.02	0.11
R	3	2	14	42	1	0.41	0.86	0.03	0.07
R	4	1	3	12	1	0.35	0.78	0.03	0.09
R	4	2	13	52	1	0.45	0.87	0.03	0.06
R	5	1	3	15	1	0.38	0.84	0.03	0.07
R	5	2	14	70	1	0.48	0.99	0.03	0.06
S(n)	5	2	14	70	0	0.48	0.0219	0.0007	0.22
S(n)	5	2	14	70	1	0.48	0.0206	0.0007	0.06
S(f)	5	2	14	70	0	0.48	0.0233	0.0007	0.22
S(f)	5	2	14	70	1	0.48	0.0215	0.0007	0.06
**KIRC**									
R	5	2	19	93	0	0.55	0.98	0.08	0.18
R	5	2	19	93	1	0.55	0.97	0.05	0.06
S(f)	5	2	19	93	0	0.55	0.07	0.0016	0.17
S(f)	5	2	19	93	1	0.55	0.06	0.0016	0.07

“Distance” of HCR/HCS from the reference data for varying parameters. Column “type” symbols: R, S(n), S(f) refer to HCR, HCS(n), HCS(f), respectively. *k*, *g*—number of *PC*s per cluster, number of levels in hierarchical clustering. $n_{C},n_{\text{PC}}$—total number of clusters, total number of *PC*s. For column +N:1 means added noise, 0 means no noise added. *V*_E_—a fraction of variance explained. *R*_mx_, *R*_avg_ are, respectively, maximal and average square of correlation coefficients computed between every *X_i_* from the original dataset and $X\prime_{j}$ from the reconstructed/simulated version. **CR—**RMSE computed between a correlation matrix computed for the original and one computed for reconstructed/synthetic data.

For brevity, for the BRCA dataset, the data for reconstructions without noise are shown only for models obtained with two principal components per cluster at each level. Trends observed here are also observed in models with a larger number of *PC*s. A full table showing parameters using more layers than 2 can be found in the Supplementary Material. As can be expected, both using multiple layers of the clustering hierarchy and using multiple *PC*s at each level improves the level of variance explained by the model, and decreases the RMSE between correlation matrices. Both actions also increase maximal correlations and decrease average correlations between original and reconstructed data. Too simple models don’t reproduce the original variables well and also contain too many weak spurious correlations. As expected, adding noise to the system decreases the level of spurious correlations and, at the same time, significantly decreases RMSE between the original correlation matrix and one computed for reconstructed/simulated data, by removing the spurious correlations. For the KIRC dataset, we report at the bottom of the table the version with g=2,k=5, the one which is also shown on plots. We note that here, the clustering was able to capture the total variance better than in the BRCA dataset (0.55 versus 0.48), and the algorithm already explained the full variance of some of the clusters (which is evident, e.g. from that total of 93 PCs was sufficient instead of 95). This is also the reason why we observe lower differences between the “noisified” and “clean” versions. The simulations of new data, as we can see in rows beginning with “S(f)” or “S(n),” are close to the real data in terms of correlation matrix on the same level as reconstructions, while their overall correlation with real data is effectively zero.

### 3.1 Correlation networks in real and synthetic datasets

The comparison of network topology summary statistics between reference, reconstructed, and simulated data is shown in Figs 2 and 3. Weights in constructed networks were based on squares of correlation coefficients. Distributions of topology descriptors for our methods are really similar inside each of the noiseless/noisified groups of methods. Here we show some of them, see Supplementary Material for complete set. The top row on both figures displays the comparison of the distribution of the clustering coefficient (*cc*) (Zhang and Horvath 2005) in between the reference data (in blue) and variants of synthetic/reconstructed data. One can immediately see that noiseless reconstructions, neither with straightforward SVD nor with our hierarchical approach, don’t agree well with the reference. The *cc*s of datasets reconstructed from the *PC*s are significantly higher than those observed in the original data. On the other hand, adding noise to the system leads to distributions that are either very close or indistinguishable. Identical behaviour is also observed for the second metric describing the properties of the correlation networks, namely the weighted degree (Zhang and Horvath 2005), displayed in the bottom row of Figs 2 and 3.

Topological descriptors of datasets obtained from noisified variants of reconstructions or HCS algorithm display behaviour identical to the reference data, while the distributions for the datasets obtained with the WGCNA simulations visibly diverge from the reference. This shows that using 1 *PC* of each cluster is not sufficient, as in the case of basic WGCNA simulation protocol.

**Figure 4.**
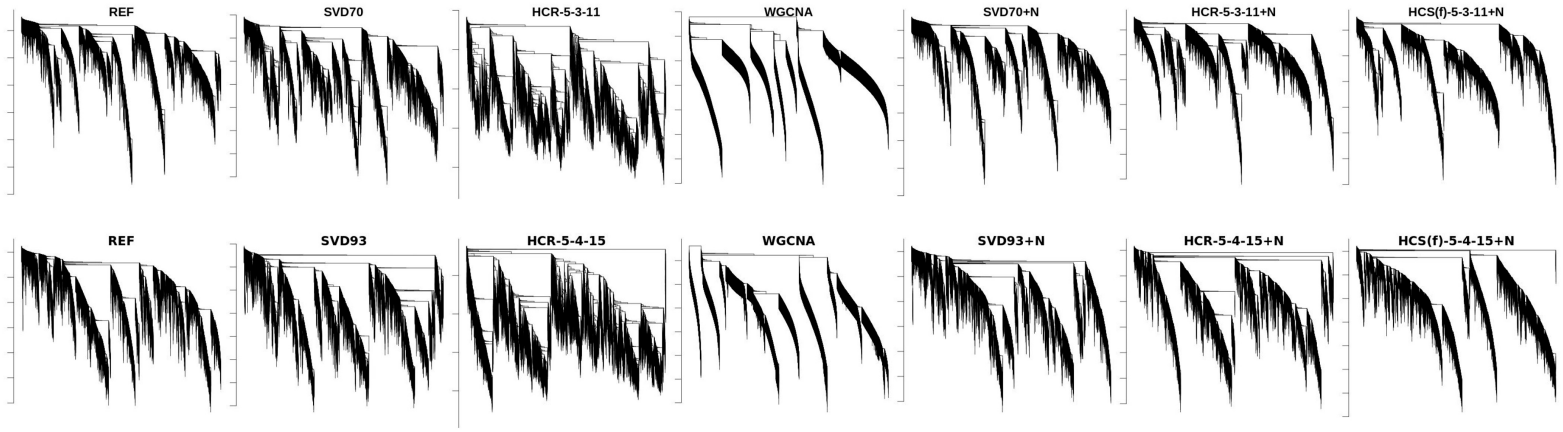
The dendrograms of the hierarchical clustering for the original data and various reconstruction and simulation schemes, for both datasets. The top row shows results on BRCA data, and the bottom one—for KIRC. REF is “reference”; other labels are as in Fig. 2. Hierarchical clustering displayed is based on topological overlap measure (Zhang and Horvath 2005) derived from squares of correlations.

The differences between methods can also be observed in the general appearance of the dendrograms arising in the hierarchical clustering of produced variables, see Fig. 4. Dendrograms for datasets obtained from reconstructions and HCS with added noise look very similar to reference, noiseless variants have visibly more rough structure, whereas simulation with WGCNA reflects the overall structure of data, but the resulting dendrogram is too smooth—lacking internal structure within individual branches.

## 4 Discussion

HCS produces faithful representations of the original datasets and allows for the generation of datasets that are not correlated with the original, yet reproduce its general structure. However, one should note, that the simulation method leads to replication of the covariance of the generating *PC*s, which is an inherently linear operation, hence we may miss non-linear relations that are present in the original data. Importantly we show that reconstruction alone, without substituting leftover variance with independent noise, is not realistic in itself. An interesting question, that is worth investigating is what ratio of signal to noise is required to get a realistic correlation network topology or what is the suspected “true” noise percentage in the overall variance and how to determine it in a statistical manner.

Our results show that the intuitive WGCNA cluster-wise PCA method indeed can be extended for building more realistic models. It brings an interesting potential for including interpretability in the faithful data simulation process. HCR and HCS, in general, can be used with any clustering method, implying that the procedures could be used not only for the validation of supervised approaches but also for assessing the degree to which a clustering algorithm can effectively capture the hierarchy of correlation structure well—one could argue that the fewer layers and clusters per fixed constraint on *PC*s one needs, the more these clusterings are descriptive of the correlation as a whole.

## Supplementary Material

btae392_Supplementary_Data

## Data Availability

All code used to produce presented results along with preprocessed input data is available at a public repository: https://github.com/p100mma/hcrs_omics. Two preprocessed datasets included there originate from online databases: BRCA data – European Genome-Phenome Archive, accesion ID: EGAS00000000083, KIRC data – The Cancer Genome Atlas: TCGA-KIRC.
