# Supplementary material for: HCS—hierarchical algorithm for simulation of omics datasets
Source: Bioinformatics. 2024 Sep 4;40(Suppl 2):ii98–ii104. doi: 10.1093/bioinformatics/btae392 (PMC11373347; doi:10.1093/bioinformatics/btae392)
Supplement: btae392_Supplementary_Data [file btae392_supplementary_data.zip › ECCB_HCRS_supplement25.05.24.pdf]

HCS- hierarchical algorithm for simulation of omics datasets: supplementary material

Piotr Stomma[1,2] Witold Rudnicki[1,2]

[1]Faculty of Computer Science, University of Bialystok, Ciolkowskiego 1M, 15-245, Poland
[2]Computational Centre, University of Bialystok, Ciolkowskiego 1M, 15-245, Poland

May 25, 2024

1 Detailed description of the HCR algorithm

1.1 Definitions

We assume that dataset is described by set of N random variables $\mathcal{X} = \{X_1, X_2 \dots X_N\}$. Each sample is an observation drawn from an N-variate distribution described by $\mathcal{X}$ and is independent of every other sample.

Each gene is associated with one index in $\mathcal{I} = \{1, 2 \dots N\}$ and each $X_i \in \mathcal{X}$ corresponds to one gene by the index i. We define clustering $P = \{C_1, C_2 \dots\}$ as a partition of indexes: $\mathcal{I} = \cup_i C_i$, where for $i \neq j : C_i \cap C_j = \emptyset$. Hierarchical clustering is a sequence of (nested) clusterings, in which the sets of the P_{i+1} subdivide clusters of the previous clustering P_i. We use a tuple-based notation to index subclusters. Tuples that index clusters in P_j are of length j and for $j \geq 2$, we allow for P_j to not cover the whole index set $\mathcal{I}$, for reasons which will be apparent later. For example, if $C_1 \in P_1$ and is divided into three clusters, then: $C_1 = C_{1,1} \cup C_{1,2} \cup C_{1,3}$ and $\forall_j : C_{1,j} \in P_2$.

$V^{\mathcal{X}}_{C_i}$ will mean sum of variances of variables X_i from $\mathcal{X}$ with $i \in C_i$, that is

$$V^{\mathcal{X}}_{C_i} = \sum_{j \in C_i} Var(X_j) \tag{1}$$

If we omit the subset of indexes in the notation, like $V^{\mathcal{X}}$, that means the sum runs over all elements of $\mathcal{X}$.

We will assume in the indexing of principal components the typical order in which as index j increases, the variance of j-th principal component decreases. Let j-th principal component of some set $\mathcal{A}$ be denoted as $PC^{\mathcal{A}}_j$. For classical

PCA, it is true that [Zou et al., 2006]:

$$X_i = \sum_j \beta_{ij} PC_j^{\mathcal{X}} \tag{2}$$

$$Var(\sum_j PC_j^{\mathcal{X}}) = \sum_j Var(PC_j^{\mathcal{X}}) = V^{\mathcal{X}} \tag{3}$$

If we limit the sum in eq. 2 to $j \leq k$, we will obtain an approximation of X_i by the first k PCs, which we will call a k-th order reconstruction of X_i.

k-th order block-wise PCA reconstruction of set $\mathcal{X}$ based on clustering P is then defined as a set of random variables $\mathcal{X}'$, where each $X'_j \in \mathcal{X}'$ is $X'_j = \sum_{l=1}^{k} \beta_{ijl}^{\mathcal{X}} PC_{il}^{\mathcal{X}}$ where:

- $j \in C_i$, $C_i \in P$ (C_i is a cluster of a variable X_j),
- $PC_{il}^{\mathcal{X}}$ is the l-th PC of all X_m with $m \in C_i$, while $\beta_{ijl}^{\mathcal{X}}$ is the corresponding coefficient used for reproducing X_j.

For indexing PC and β, an additional index i is needed to signify the cluster C_i. To use hierarchical clusterings in the above notation, we have to substitute for one index i a tuple of indexes J. For example, for a cluster in $C_J \in P_4$, length of J is 4 and k-th principal component of $\mathcal{X}$ constrained to C_J is given by a expression $PC_{Jk}^{\mathcal{X}}$.

The following statements are true in the settings of the definitions just described. See the next section for derivation in the general case of HCR.

Call a elements of a set $\mathcal{E}' = \{X_i - X'_i | i \in \mathcal{I}\}$ residuals of the reconstruction $\mathcal{X}'$. Let $E'_i = X_i - X'_i$ (E_i is the i-th residual). $Var(E'_i) = Var(X_i) - Var(X'_i)$. Assume that in constructing the reproduction of E_i we have not used all of the principal components. Then $Var(E'_i) > 0$. Moreover, residuals $\mathcal{E}'$ and reproduced variables $\mathcal{X}'$ located in the same cluster are uncorrelated. For variables in the same cluster, principal components of residuals are also uncorrelated with principal components used for constructing $\mathcal{X}'$.

1.2 The HCR Algorithm

In the proposed approach, input parameters are:

- limiting fraction of variance to explain: $f_E \in [0, 1]$,
- k - a constraint on the maximum number of PCs to use per cluster,
- two clustering methods for random variables based on their correlation strength: an initial "adaptive" clustering algorithm and a "separator" algorithm.

The initial adaptive algorithm should be able to detect clusters of various sizes, and preferably should be able to perform initial prefiltering: it should classify variables distant to every other variable as "noise" (not real clusters).

The "separator" algorithm is used to further subdivide "high-confidence" clusters produced by the initial clustering, based on the correlation of the residuals. The separator method should produce a complete partition of a set of objects. It is used further to generate subdivisions at lower levels: $P_2, P_3 \dots$.

The main algorithm consists of one necessary step: initial division, and then a certain number of repeats of the subdivision step. The algorithm repeatedly checks if there is still some unexplained variance left in each of the (sub)clusters. We will call a cluster unsaturated if it meets these conditions.

1.2.1 Initial division step

Suppose $P_1 = \{C_0, C_1 \dots\}$ where C_0 is the "noise" part of the dataset. Let $C_{i\neq 0} := \cup_{i\neq 0} C_i$ be the union of "non-noise" clusters. If $V^{\mathcal{X}}_{C_{i\neq 0}} < f_E V^{\mathcal{X}}$, then stop – there is not enough variance in the noiseless part of the clustering to obtain target fraction f_E. Otherwise calculate f such that $fV^{\mathcal{X}}_{C_{i\neq 0}} = f_E V^{\mathcal{X}}$. For $j \in C_{i\neq 0}$, calculate variables $X^1_j = \sum_{l=1}^{k_i} \beta^{\mathcal{X}}_{ijl} \, PC^{\mathcal{X}}_{il}$, where for particular j, $j \in C_i$ and $k_i = \min(k, k^f)$, where k^f is minimum number of PCs of C_i that will explain $fV^{\mathcal{X}}_{C_i}$.

Since initial division allows some variables in $\mathcal{X}$ to be left over in C_0, to keep the notation simple, we set $X^1_i = 0$ if $i \in C_0$.

1.2.2 Subdivision step in general

Let $\mathcal{X}^g$ be reconstruction of the previous residuals $\mathcal{E}^{g-1}$ (or $\mathcal{X}$ if $g = 1$), and let $\mathcal{E}^g$ be residuals of $\mathcal{X}^g$. Assume $\mathcal{X}^g$ was based on partition P_g of clusters C_K indexed by g-element tuples.

Assess the saturation of each cluster (by checking how many PCs it is required to explain its leftover part of the variance, in comparison with k). Do the next steps for each of the unsaturated clusters C_K. Based on the residuals $\mathcal{E}^g$, subdivide C_K into clusters C_L, indexed by $g+1$ element tuples L. Calculate the unexplained variance in C_K that is left to explain:

$$target_{C_K} := fV^{\mathcal{X}}_{C_K} - \sum_{j=1}^{g} V^{\mathcal{X}^j}_{C_K} \tag{4}$$

Calculate k-th order reconstruction of residuals $\mathcal{E}^g$ which belong to C_K. based on clusters C_L:

- calculate fraction of variance of residuals in C_K that will suffice: $f_K \in [0, 1]$ for which $f_K V^{\mathcal{E}^g}_{C_K} = target_{C_K}$
- for each E^g_i with $i \in C_K$, calculate its reconstruction:

$$X^{g+1}_i := \sum_{l=1}^{k_L} \beta^{\mathcal{E}^g}_{Lil} PC^{\mathcal{E}^g}_{Ll} \tag{5}$$

  where $i \in C_L$, $k_L = \min(k, k^L_f)$ and k^L_f is the required number of $PC^{\mathcal{E}^g}_{Ll}$ to explain $f_K V^{\mathcal{E}^g}_{C_L}$.

Before executing the next step, put all subclusters C_L of unsaturated clusters C_K into P_{g+1}.

If one wants to stop at this point, final reproduction is a set of random variables $\mathcal{X}^R = \left\{\sum_{l=1}^{g+1} X_i^l | i \in \mathcal{I}\right\}$. Since some of the clusters were explained to the desired accuracy by reconstruction on the previous g-th level, X_i^{g+1} that corresponded to i in the already saturated cluster were actually not calculated. To keep the notation simple, we assume that $X_i^{g+1} = 0$ if $i \in C_J$, where $C_J \in P_h, h \leq g$ and C_J is saturated (or like if in initial division, $i \in C_0$).

1.3 Covariances of reconstructions and residuals

1.3.1 Covariance of reconstruction and residual at one level of hierarchy in the same cluster

Suppose $i, j \in C_{I_n} \subset C_{I_{n-1}} \subset \ldots C_{I_2} \subset C_{I_1}$, with each $C_{I_j} \in P_j$. First we show that E_i^n and X_j^n are uncorrelated.

Variable X_j^n is a linear combination of k PCs of the set $\{E_l^{n-1} | l \in C_{I_n}\}$, while E_i^n is a linear combination of the remaining ones:

$$X_j^n = \sum_{h \leq k} \beta_{I_n jh}^{\mathcal{E}^{n-1}} PC_{I_n h}^{\mathcal{E}^{n-1}} \tag{6}$$

$$E_i^n = \sum_{h > k} \beta_{I_n ih}^{\mathcal{E}^{n-1}} PC_{I_n h}^{\mathcal{E}^{n-1}} \tag{7}$$

Then the result is immediate:
$cov(E_i^n, X_j^n) = \sum_{h \leq k} \beta_{I_n jh}^{\mathcal{E}^{n-1}} \sum_{g > k} \beta_{I_n ig}^{\mathcal{E}^{n-1}} cov(PC_{I_n g}^{\mathcal{E}^{n-1}}, PC_{I_n h}^{\mathcal{E}^{n-1}}) = 0$, since for each term of the sum $g \neq h$.

1.3.2 Covariance of residual and lower level deconstruction of X_i

Now we will show how to derive $cov(E_i^n, X_i^m) = 0$ for $m < n$. To see this, one has to substitute in the expression $cov(E_i^n, X_i^m)$ for the residual E_i^n its expansion in terms of principal components, and then one has to substitute residuals of the previous level for the principal components. E_i^n is a linear combination of PCs of residuals of the previous level as we have noted above. Principal component of a set $\mathcal{A}$ is a linear combination of all variables in $\mathcal{A}$, so $PC_{I_n h}^{\mathcal{E}^{n-1}} = \sum_{i_n \in I_n} \gamma_{i_n}^h E_{i_n}^{n-1}$ for some coefficients $\gamma_{i_n}^h$. Then each $E_{i_n}^{n-1}$ is a linear combination of a portion of PCs of the set $\{E_l^{n-2} | l \in I_{n-1}\}$. So we can substitute each residual with the linear combination of PCs of the cluster of the previous partition, and each such PC can be then substituted with a linear combination of the residuals of the previous reconstruction. If we continue substitutions, in the end, we will reach an expression which is a linear combination of terms $cov(E_j^m, X_i^m)$, where j, i belong to to I^m, if we factor out coefficients and sums. Each such term satisfies the condition of the first relation we have shown, so each covariance term is 0. Therefore $\forall p \leq n : cov(E_i^n, X_i^p) = 0$.

Similarly we can derive that $cov(X_i^m, X_i^n) = 0$ for $m < n$. Since X_i^n is a linear combination of PCs of set $\mathcal{E}^{n-1}$, it can be expressed as some linear combination of $\{E_l^m | l \in I_m\}$ in a chaining manner like above, so the covariance is a linear combination of terms $cov(X_i^m, E_j^m), j \in I_m$. X_i^m, E_j^m satisfy the condition in the first relation we have proved, so those covariances are all zero.

1.3.3 Total variance of X_i

In that manner, we see that $Var(X_i) = Var(E_i^n) + \sum_{j=1}^n Var(X_i^j)$

2 HCS Algorithms

As an input, one provides HCD of a real dataset and additionally for procedure HCS(f) – a family of parametric distributions, which will be fitted to PCs from HCD. We will simplify the notation here. Supposing there are M PCs in HCD in total, they will be marked here just by $PC_1, PC_2 \ldots PC_M$, and associated coefficients of linear combinations to use for reconstruction of X_j are then just β_{ji}. Note that for PCs coming from different (sub)clusters, their correlations might be nonzero.

We remind that g denotes the number of partitions used in input decomposition. HCS(n) works as follows:

1. Estimate $M \times M$ covariance matrix Σ of input PCs.
2. Generate a desired number of samples from M-variate normal distribution using Cholesky decomposition of Σ. Denote obtained variables as $Z_1, Z_2 \ldots Z_M$, where $cov(Z_i, Z_j) = \Sigma_{ij}$.
3. Generate the simulated variables $X_j^S = \sum_i \beta_{ji} Z_i$, where sum runs over all indexes of PCs of clusters and subclusters to which j belongs.
4. (Optionally) To each of X_j^S, add random noise from $\mathcal{N}(0, \sigma_j^S)$ with $\sigma_j^S = SD(E_j^g)$.
5. Adjust means of X_j^S, such that $E(X_j^S) = E(X_j)$.

Constraint on the number of PCs one can use here comes from Cholesky decomposition (see Introduction). If a number of variables exceed the number of observations only by a small amount, it might be reasonable to consider some of the mentioned methods of "repairing" a non-positive definite matrix [Higham, 2002]. This option was not explored in this research.

Without an optional step, one simulates a version of the original data where main correlations are amplified. This optional step makes the variance of each X_j^S to be equal to $Var(X_j)$ and lowers the overall correlation strength to a realistic level – as our empirical evidence shows.

Steps of HCS(f) are listed below:

1. To each PC_i, fit a parametric distribution with computable (or approximable) c.d.f. F_i and quantile function F_i^{-1}.
2. Calculate representations of PCs under normal distribution: $H_i := (\Phi^{-1} \circ F_i)(PC_i)$, where Φ^{-1} is standard normal quantile function.
3. Do steps 1 and 2 of the procedure I, but for H_i in place of PC_i. Standardize resulting variables Z_i.
4. Do step 3 of proc. I, but with replacing Z_i by $PC_i^S = (F_i^{-1} \circ \Phi)(Z_i)$, where Φ is standard normal c.d.f.
5. Do steps 4 and 5 from procedure I.

3 Comparison details

Below details of the parameters used apply to both tested datasets (BRCA and KIRC), unless explicitly stated otherwise.

3.1 HCR/HCS

For the initial division in our method, we have chosen the Markov Clustering Method (MCL) with the weight matrix of the graph set to the square of the Pearson correlation. The connections that corresponded to correlations that were not statistically significant at 0.05 level (after Holm correction) were set to zero. Inflation (granularity control parameter) was set to a conservative value of 2. Clusters significantly smaller than the rest were marked as "noise", which left 64 variables "clusterless" in the breast cancer data and 626 for KIRC.

For the further subdivisions, we tested candidate splits into a number of clusters ranging from 2 to 7. We have used just one additional subdivision (levels of hierarchy g=2) and $k = 5$ PCs per (sub)cluster.

Lastly, in HCS(f), for fitting metalog distributions, we have utilized `R` package `rmetalog` [Faber and Jung], with the same settings used for each of the PCs – an unbounded variant of the distribution with a number of terms set to 5 and step length to 0.01.

For the KIRC data, for one principal component, namely 1st of the cluster number '2' in the first layer, we set the number of terms to the value of 10. The reason for this is that the distribution of this particular variable was bimodal, and a simpler form of the metalog distribution could not catch that detail, resulting in an underestimated variance. Without such intervention, the mode of the distribution of the clustering coefficient of simulated data was unacceptably low (in comparison to the reference). This makes sense because cluster number '2' contains around 8 thousand genes, so its 1st PC accounts for big chunk of the total variance. Such observation emphasizes the importance of checking (for the HCS(f) method) if the variance of the main components of the biggest clusters is not underestimated in case the end result of the simulation is not satisfactory. Checking the fit of distributions of generating PCs is thus advised.

3.2 WGCNA details

For producing an input clustering to the WGCNA [Zhang and Horvath, 2005] simulation, we followed a protocol in which one first transforms a correlation matrix into a similarity matrix S by raising absolute values of all of its elements to a power: $S_{ij} = |\rho_{ij}|^p$, where $p \geq 1$, and ρ_{ij} is Pearson correlation coefficient of X_i and X_j. The value of p is chosen to induce a so-called approximate scale-free topology in the weighted graph with weight matrix S, which is evident as a linear relationship in the histogram plot of weighted degree (called Connectivity in WGCNA approach) in the log-log scale. Then one uses average linkage hierarchical clustering, based on a derived robust measure called topological overlap, defined in terms of initial S. Finally, clusters from such a dendrogram

are obtained by using a Dynamic Tree Cut method, which allows for classifying some of the genes as "clusterless", or "noise".

In the construction of the network, we have used a value of power $p = 5$, and for the Dynamic Tree Cut method, we have chosen a "hybrid" variant with an additional PAM stage in the end, setting the minimum cluster size to 90 and "deepSplit" parameter (controlling granularity) to 4 (for BRCA data). Parameters used in the KIRC dataset differ only by the minimum cluster size, which was set to 150.

3.2.1 Further details of comparison fairness

Sets of variables deemed as "clusterless" are not modelled either by our simulation or WGCNA based one. However, those "clusterless" sets differ between used clustering methods as a basis for simulations. To simplify comparison, we omit "clusterless" variables in the calculation of the metrics. This gives an advantage in that aspect to the method, which has found a bigger "clusterless" zone (less conditions to replicate). For the plain SVD-based reconstructions, we compute metrics on the whole set of variables available. In general, we note that the original simulation function, as implemented in the WGCNA package, was unlikely to be used for simulating data at the scope we want to achieve. In this method, to simulate variable X_i, one computes $X'_i := PC_1(X_i) + \epsilon_i$, where $PC_1(X_i)$ is the 1st PC of the cluster to which variable X_i belongs and ϵ_i is a noise term ensuring that $cor(X_i, PC_1(X_i))^2 \approx cor(X'_i, PC_1(X_i))^2$. Original simulation function models sorted squared values of correlations of members of a cluster with their 1st PC by an approximate curve (either concave, convex or straight line), running from minimum to maximum value of such quantities. We have found that such approximation fits poorly – sorted values of such correlations seem to follow an 'S' pattern. Original WGCNA simulation was also unlikely suited to model marginal distributions exactly. Therefore, to make the comparison more fair, we compare against a slightly tweaked version, which performs better in our comparison than the original implementation. A straightforward fix that we have implemented is to use the simulation-defining equation directly, using the exact estimate of the correlation coefficient, instead of its approximate value obtained from a poorly fitted curve. Also, at the end of the method, we scale each synthetic variable such that its mean and variance correspond to the one in the reference dataset.

4 Reconstruction and simulation: distance to the reference – comparison table

Table 1: Reconstruction accuracy for varying parameters of the model

k	g	n_C	n_{PC}	+N	$\frac{V^{\mathcal{X}'}}{V^{\mathcal{X}}}$	$cor(X_i, X'_j)^2$		**CR**
						max	mean	
Reconstructions (HCR) of BRCA for varying values of k, g								
2	1	3	6	0	0.27	0.86	0.09	0.44
2	2	14	28	0	0.37	0.94	0.08	0.33
2	3	64	128	0	0.46	0.99	0.07	0.27
2	4	256	512	0	0.56	1	0.06	0.21
2	1	3	6	1	0.27	0.74	0.02	0.12
2	2	14	28	1	0.37	0.87	0.03	0.09
2	3	64	128	1	0.46	0.99	0.03	0.07
2	4	256	512	1	0.56	1	0.03	0.07
3	1	3	9	1	0.32	0.78	0.02	0.11
3	2	14	42	1	0.41	0.86	0.03	0.07
3	3	61	183	1	0.51	1	0.03	0.06
3	4	227	681	1	0.61	1	0.03	0.05
4	1	3	12	1	0.35	0.78	0.03	0.09
4	2	13	52	1	0.45	0.87	0.03	0.06
4	3	61	244	1	0.55	1	0.03	0.05
4	4	245	980	1	0.65	1	0.03	0.05
5	1	3	15	1	0.38	0.84	0.03	0.07
5	2	14	70	1	0.48	0.99	0.03	0.06
5	3	57	285	1	0.56	0.99	0.03	0.05
5	4	168	840	1	0.65	1	0.03	0.04
HCS(n) (BRCA)								
5	2	14	70	0	0.48	0.0219	0.0007	0.22
5	2	14	70	1	0.48	0.0206	0.0007	0.06
HCS(f) (BRCA)								
5	2	14	70	0	0.48	0.0233	0.0007	0.22
5	2	14	70	1	0.48	0.0215	0.0007	0.06
HCR (KIRC)								
5	2	19	93	0	0.55	0.98	0.08	0.18
5	2	19	93	1	0.55	0.97	0.05	0.06
HCS(f) (KIRC)								
5	2	19	93	0	0.55	0.07	0.0016	0.17
5	2	19	93	1	0.55	0.06	0.0016	0.07

Top section: comparison of the accuracy of HCR reconstructions on the BRCA dataset, for varying k, g. Bottom sections: similar metrics computed for both HCS methods shown in the first experiment (BRCA) and for HCR constructed during additional test on KIRC data. k, g- number of PCs per

cluster, number of levels in hierarchical clustering; n_C, n_{PC} - total number of clusters, total number of PCs; $+N$ set to 1 means version with added noise, 0 means no noise added; $\frac{V^{\mathcal{X}'}}{V^{\mathcal{X}}}$ is fraction of variance explained; $cor(X_i, X_j')^2$ is square of correlation coefficient computed between every X_i from original dataset and X_j' from reconstructed/simulated version and columns show its max and mean value; **COR** RMSE is computed between a correlation matrix computed for the original and one computed for reconstructed/synthetic data.

5 Correlation network characteristics – comparison plots

5.1 Distributions of topology descriptors

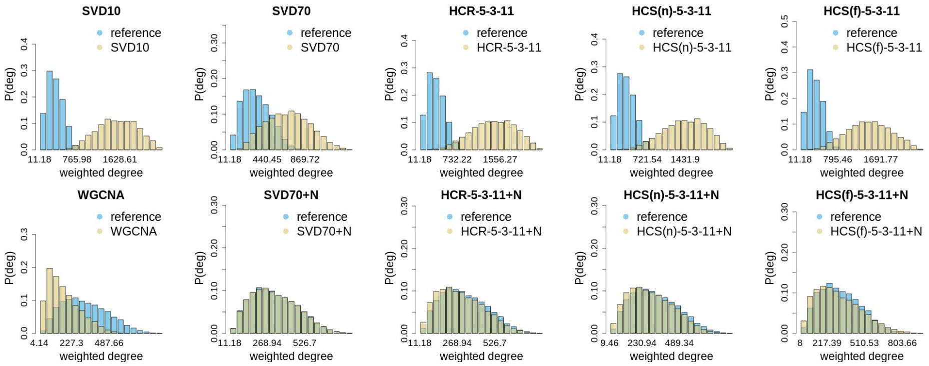

Figure 1: The weighted degree distributions for different methods of reconstruction and simulation of the BRCA dataset (yellow histograms). The distribution for the original dataset (blue histograms) is shown for comparison. The labels are as in Figure 2 of the main manuscript. Similar labels and colours are used for further plots in this subsection.

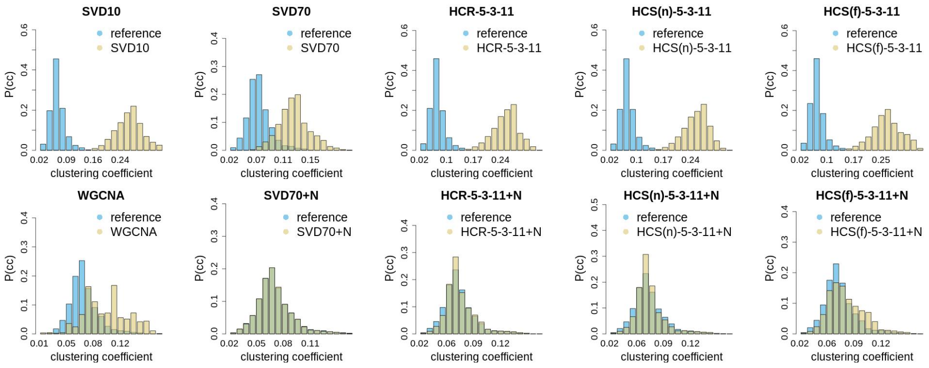

Figure 2: Distributions of clustering coefficient, BRCA dataset.

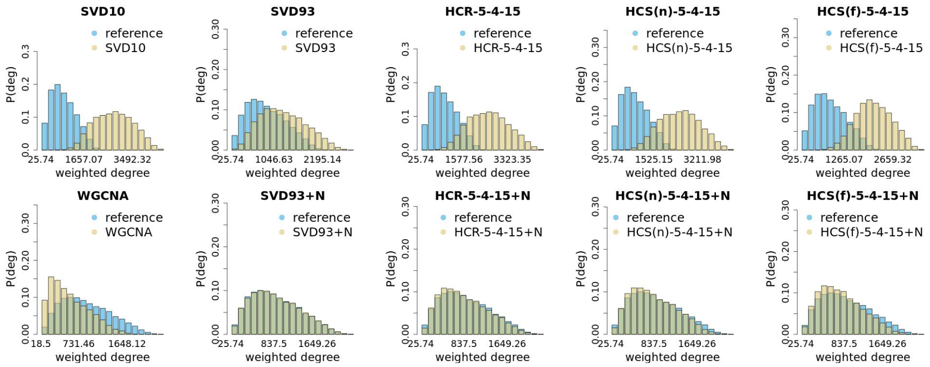

Figure 3: Weighted degree, KIRC dataset.

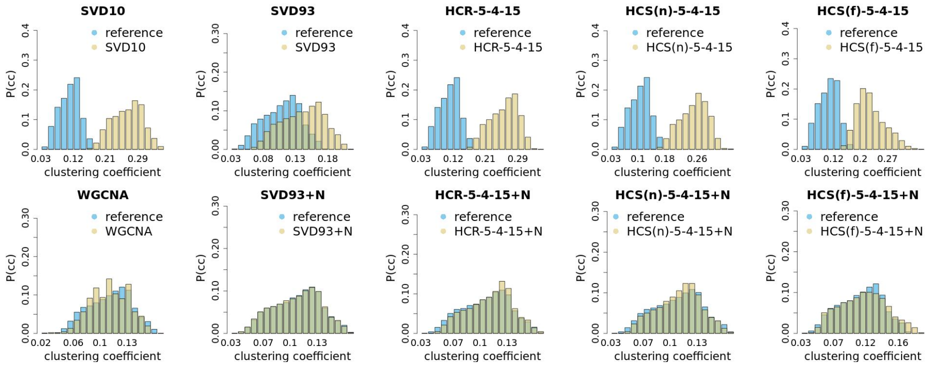

Figure 4: Clustering coefficient, KIRC dataset.

6 KS distances between simulated variables and corresponding real distributions

We have computed KS distance [Dodge, 2008] between each synthetic and real variable. We used that statistic to quantify differences between empirical c.d.f's (*ecdfs*) of synthetic and real data:

$$KS_d(X_i, X_i') = \max |F_{X_i}(X_i') - F_{X_i'}(X_i')| \tag{8}$$

where X_i is the reference variable, X_i' is the simulated one, F_{X_i} is *ecdf* of X_i and $F_{X_i'}$ is *ecdf* of X_i'.

Table 2 shows how the summary statistics of that metric look across all variables in the dataset. We see that PCs in real data do not necessarily follow a normal distribution, and distances between fitted versions (HCS(f)) are smaller.

For comparisons between surface variables, differences between approaches are negligible. Some of the variables do not fit well by either of the procedures, as seen by the maximum value of the metric.

Table 2: Summary statistics of KS distances between synthetic and reference variables for BRCA data

	Min.	Q1	Q2	Q3	Max.
*PC*s					
HCS(n)	0.02	0.03	0.04	0.05	0.14
HCS(f)	0.01	0.02	0.02	0.03	0.05
simulations					
WGCNA	0.01	0.03	0.05	0.07	0.4
SVD: 70 PCs + noise	0.01	0.03	0.04	0.06	0.33
SVD: 10 PCs + noise	0.01	0.03	0.05	0.07	0.37
hier.rec. + noise	0.01	0.03	0.05	0.07	0.38
procedure I + noise	0.01	0.04	0.05	0.08	0.37
procedure II + noise	0.01	0.03	0.05	0.08	0.38

Upper section: KS distances calculated between real and synthetic principal components.
Bottom section: KS distances between variants of (noisified) simulations.

References

Y. Dodge. *The Concise Encyclopedia of Statistics*. Springer New York, New York, NY, 2008.

I. Faber and J. Jung. rmetalog: The metalog distribution. `https://CRAN.R-project.org/package=rmetalog`. (10.02.2024, date last accessed).

N. Higham. Computing the nearest correlation matrix—a problem from finance. *IMA Journal of Numerical Analysis*, 22(3):329–343, 2002.

B. Zhang and S. Horvath. A general framework for weighted gene co-expression network analysis. *Statistical Applications in Genetics and Molecular Biology*, 4, 2005.

H. Zou, T. Hastie, and R. Tibshirani. Sparse principal component analysis. *Journal of Computational and Graphical Statistics*, 15(2):265–286, 2006.
